# The safety and effectiveness of chenodeoxycholic acid treatment in patients with cerebrotendinous xanthomatosis: two retrospective cohort studies

**DOI:** 10.1007/s10072-019-04169-8

**Published:** 2019-12-20

**Authors:** Aad Verrips, Maria Teresa Dotti, Andrea Mignarri, Bianca M. L. Stelten, Sue Verma, Antonio Federico

**Affiliations:** 1 Canisius-Wilhelmina Hospital, Nijmegen, the Netherlands; 2 Department of Medicine, Surgery and Neuroscience, University of Siena, Siena, Italy; 3 Catharina Hospital, Eindhoven, the Netherlands; 4 Leadiant Biosciences Ltd., London, UK

**Keywords:** Chenodeoxycholic acid, Cerebrotendinous xanthomatosis, Metabolic disorders, Italy, The Netherlands

## Abstract

**Objective:**

To evaluate the safety and effectiveness of chenodeoxycholic acid (CDCA) treatment in patients with cerebrotendinous xanthomatosis (CTX).

**Methods:**

Two retrospective cohort studies were conducted in CTX patients who underwent CDCA treatment: one in the Netherlands (NL; CDCA-STUK-15-001) and one in Italy (IT; CDCA-STRCH-CR-14-001). Eligible patients were aged 2–75 years, had been diagnosed with CTX, and were treated with CDCA orally for ≥1 year. The impact of CDCA treatment on biochemical markers (including serum cholestanol levels) and disease signs and symptoms were assessed, in addition to the safety and tolerability of CDCA treatment.

**Results:**

A total of 35 patients were screened in the NL study and were diagnosed with CTX at 25.6 (± 13.7 SD) years on average. These patients were treated with CDCA and followed up for a median of 9.00 (range: 0.4–26.3) years. In addition, 28 patients were enrolled in the IT study and were diagnosed at 35.0 (± 11.4 SD) years on average (median duration of CDCA treatment: 5.75 [range: 0.0–25.0] years). Signs and symptoms of disease resolved, improved, or remained stable in many patients, with concomitant improvements in biochemical marker levels (serum cholestanol, *p* < 0.001; 7α-hydroxy-4-cholesten-3-one, *p* < 0.001 [IT study]).

**Conclusions:**

The outcomes of these retrospective cohort studies indicate that CDCA is effective in the long-term treatment of CTX, with an acceptable safety profile.

**Electronic supplementary material:**

The online version of this article (10.1007/s10072-019-04169-8) contains supplementary material, which is available to authorized users.

## Introduction

Cerebrotendinous xanthomatosis (CTX; OMIM 213700) is a rare, autosomal recessive disorder caused by mutations in *CYP27A1*, which lead to a deficiency in sterol 27-hydroxylase [1, 2]. The latter is important for primary bile acid synthesis, specifically the conversion of cholesterol to chenodeoxycholic acid (CDCA) and cholic acid [2, 3]. A deficiency in sterol 27-hydroxylase leads to reduced levels of CDCA; however, patients may have unaffected or higher levels of cholic acid [2, 4]. In addition, CTX patients have high levels of plasma cholestanol, which accumulates in bile and various tissues, particularly the brain [2].

Manifestations of CTX can vary, not only within families but even between twins [5]. These include prolonged neonatal jaundice, infantile-onset chronic diarrhoea, juvenile cataracts, tendon xanthomas with onset during adolescence or young adulthood, osteoporosis, and progressive psychiatric and neurological impairment [2, 6]. The latter may include mental retardation, cerebellar syndrome, pyramidal syndrome, peripheral neuropathy, epileptic seizures and dementia [2]. The accumulation of cholestanol is thought to be responsible for these neurodegenerative symptoms [2]. Progressive neurological and psychiatric dysfunction are the most disabling features of CTX [4].

More than 300 patients have been diagnosed with CTX worldwide, although the condition is likely to be underdiagnosed [2]. The estimated prevalence of CTX in white populations is 1:50,000; however, this is limited to patients with the R362C *CYP27A1* mutation [7]. The diagnosis of CTX typically involves biochemical testing for plasma cholestanol levels, which in CTX are usually markedly increased [2]. The normal range for cholestanol is 3.37 ± 1.55 mg/L (8.66 ± 3.98 μmol/L), but in CTX patients it may be several times higher than this [8–10]. Elevated plasma concentration of the bile acid precursor 7α-hydroxy-4-cholesten-3-one has also been suggested as a new diagnostic marker of CTX, although these tests are not widely available [8, 11]. Urinary bile alcohol levels can also be used for diagnosis, although genetic testing for mutations in the *CYP27A1* gene is the gold standard and is generally used for confirmation of diagnosis [2, 8].

CDCA has become the standard of care for CTX patients [4]. It prevents the accumulation of cholestanol by inhibiting bile acid synthesis through a negative feedback pathway [12]. Exogenous CDCA has been shown to improve both biochemical and clinical outcomes in CTX patients [4]. Given the natural course of CTX, the primary aim of treatment is stabilisation or improvement of neurological signs and symptoms [4]. Nevertheless, a delay in diagnosis and treatment can lead to a worse prognosis, irreversible neurological damage and severe disability [13].

A recent study by Stelten et al. (2018) demonstrated that starting treatment at an early age can reverse and prevent neurological symptoms [14]. The authors reported results from a retrospective study performed in the Netherlands that evaluated the effect of CDCA on disease progression in 56 CTX patients. Disease progression was measured through correlation between age at diagnosis and clinical characteristics, and with the course of modified Rankin Scale and Expanded Disability Status Scale (EDSS) scores at follow-up [14].

Here, we present data from two retrospective studies, submitted for regulatory purposes to the EMA, in CTX patients who underwent CDCA treatment. The aims of both studies were to evaluate the impact of CDCA on serum cholestanol levels (among other biochemical markers), the effectiveness of this treatment for ameliorating the signs and symptoms of disease, and the impact on disability scores over time. We also retrospectively evaluated the safety and tolerability of CDCA treatment in CTX patients. The results presented herein include data from 35 patients in the previously described Stelten et al. (2018) study, conducted in the Netherlands, along with results from a similar Italian retrospective study [14].

## Methods

Data are reported from two retrospective, single-centre, cohort studies, conducted in the Netherlands (‘NL’; CDCA-STUK-15-001) and Italy (‘IT’; CDCA-STRCH-CR-14-001).

### The NL study

This study was carried out at Canisius-Wilhelmina Hospital in the Netherlands, an official reference centre for CTX patients. Eligible patients were aged 2–75 years, with diagnosed CTX of ≥ 1-year duration that had been treated with CDCA (750 mg/day or 15 mg/kg/day) orally for ≥ 1 year. This study used commercially formulated CDCA, which had undergone production via an industrial process, with quality control performed on every batch.

Patients were required to have ≥ 1 cholestanol and/or urinary bile alcohol assessment ≤ 3 months prior to treatment with CDCA and ≥ 1 such value within 2 years from the beginning of therapy with CDCA. Demographic and clinical data were collected prior to CDCA treatment (baseline) and at two post-treatment visits (V1 and V2). To document the patient’s current clinical condition (CCC), the most recent available evaluation was also captured during the CCC visit. The first patient visit was on 27 November 1981 and the last on 3 June 2015. Only those patients who were able to attend hospital visits were included in the analyses presented here.

Baseline characteristics were extracted from medical records. Effectiveness analyses were conducted in the evaluable population, which included all eligible patients with (a) ≥ 1 quantitative cholestanol assessment the same day or before CDCA initiation, and ≥ 1 after initiation, or (b) ≥ 1 qualitative assessment for urinary bile alcohols the same day or before CDCA initiation, and ≥ 1 after initiation. Effectiveness variables included serum cholestanol, urinary bile alcohols and presence, absence or change in disease signs and symptoms (including psychiatric, cognitive and neurological impairment, epilepsy, polyneuropathy and diarrhoea). The latter were determined through physical examination. Additionally, neurological disability scores (Rankin Scale and EDSS) were reported.

Safety variables were collected in the safety population, which included all eligible patients who had received ≥ 1 dose of CDCA, regardless of the overall duration of CDCA treatment (i.e. < 1 or ≥ 1 year). Safety variables included adverse events (AEs; coded using MedDRA 17.0), serious AEs and laboratory tests.

### The IT study

This study was carried out at the Operative Unit of Neurology and Neurometabolic Disorders, University of Siena, Italy. Eligible patients were aged 2–75 years, with diagnosed CTX of ≥ 1-year duration that had been treated with CDCA (750 mg/day) orally for ≥ 1 year. Due to limited availability of commercially produced CDCA prior to licensing, a local pharmacy-produced galenic formulation of CDCA was used in this study.

Patients were required to have ≥ 1 cholestanol assessment and ≥ 1 routine laboratory evaluation ≤ 3 months prior to treatment with CDCA and ≥ 1 of each within 2 years of treatment initiation. Patients undergoing treatment with other bile acids, statins or steroids ≤ 3 months before diagnosis, and throughout the follow-up period, were excluded from the study. As in the NL study, demographic and clinical data were collected at baseline, V1, V2 and the CCC visit. The first patient visit was on 3 November 1988 and the last on 22 October 2014.

Baseline characteristics were collected in the screened population by extracting information from medical records. Effectiveness variables were measured in the evaluable population (all patients with ≥ 1 cholestanol assessment prior to, and ≥ 1 following, CDCA initiation). Effectiveness variables included serum cholestanol and 7α-hydroxy-4-cholesten-3-one and presence, absence or change in disease signs and symptoms (including psychiatric, cognitive and neurological impairment and diarrhoea). The latter were determined through physical examination. Additionally, neurological disability scores (Rankin Scale, EDSS) were reported.

Safety variables were measured as per the NL study.

### Statistical analyses (both studies)

Please refer to the Supplemental Material (**Online Resource**).

## Results

### The NL study

#### Patient disposition and baseline characteristics

**Fig. 1:**
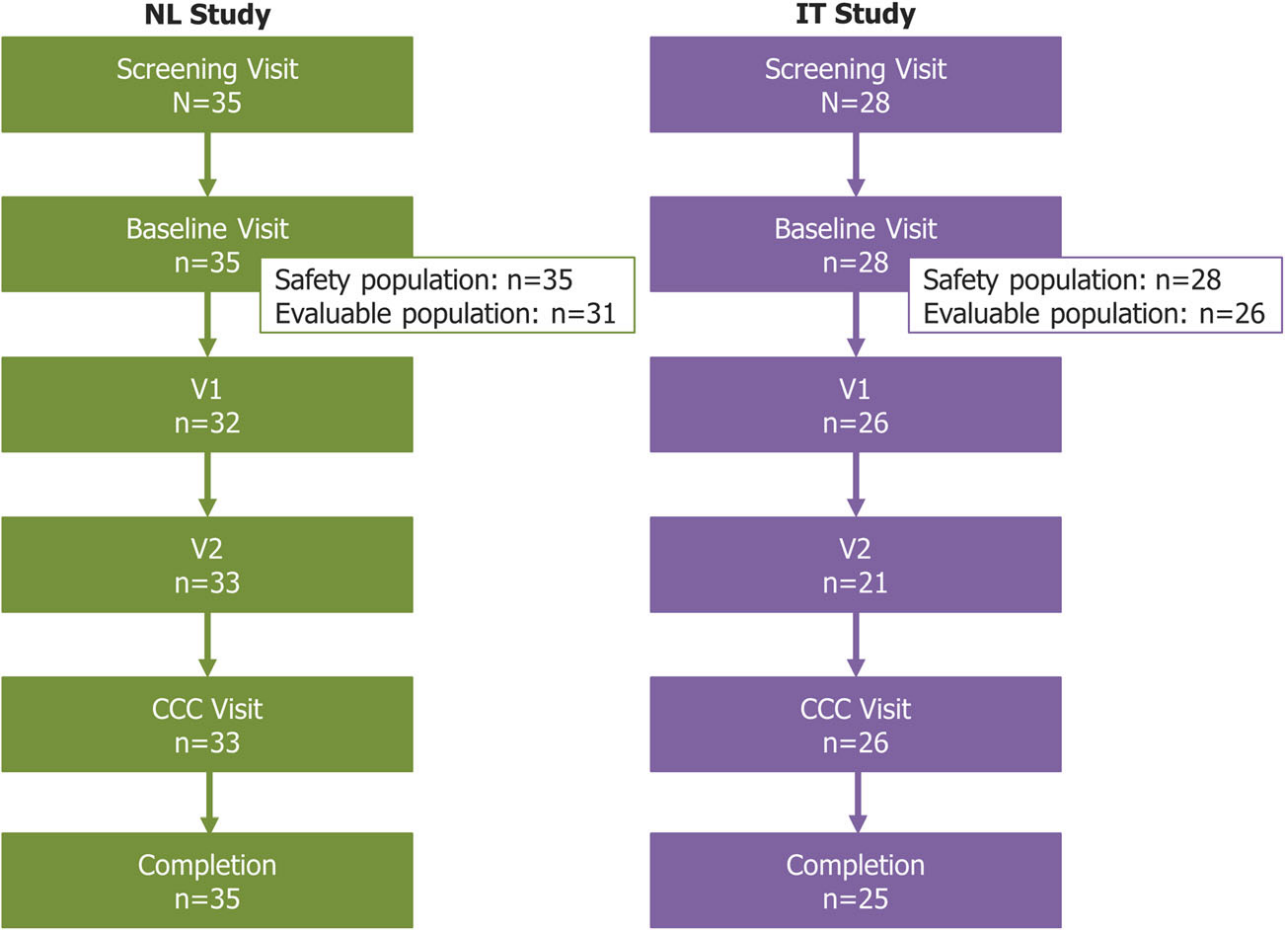
Patient disposition. Three patients (10.7%) in the IT study discontinued, either due to adverse events (n=1) or withdrawal of consent (n=2). Abbreviations: CCC: Current clinical condition; IT: Italy; NL: the Netherlands; V1: Post-treatment visit 1; V2: Post-treatment visit 2

Thirty-five patients were screened in the study, all of whom formed the safety population and 31 (88.6%) the evaluable population (Fig. 1). The majority of patients were male (n=21; 60.0%), with a mean (± SD) age of 36.6 (± 16.8) years (Table 1). The median time from baseline visit to V1 was 9.9 months (range: 1.1–186.9), to V2 was 37.6 months (9.5–252.0), and to the CCC visit was 120.9 months (26.2–305.3).

**Table 1:** Baseline demographics and clinical characteristics

	NL study (*N* = 35)	IT study (*N* = 28)
Demographics		
Mean age at disease onset, years (± SD)	–^[a]^	11.0 (± 11.2)
Mean age at diagnosis, years (± SD)	25.6 (± 13.7)	35.0 (± 11.4)
Mean age at treatment initiation, years^[b]^ (± SD)	25.8 (± 14.0)	35.0 (± 11.4)
Mean age at study initiation, years (± SD)	36.6 (± 16.8) [*n* = 33]^[c]^	47.4 (± 13.2)
Male, n (%)	21.0 (60.0)	13.0 (46.4)
Clinical characteristics^[d]^		
Mean serum cholestanol levels, μmol/L (± SD)	76.5 (± 39.0) [*n* = 27]^[e]^	87.8 (± 39.2)
Mean 7α-hydroxy-4-cholesten-3-one level, mmol/L (± SD)	–^[f]^	9.3 (± 5.7) [*n* = 16]^[g]^
Neurological impairment present, n (%)	20 (64.5)	20 (76.9)
Psychiatric impairment present, n (%)	6 (19.4)	13 (50.0)
Cognitive impairment present, n (%)	18 (58.1)	20 (76.9)
Diarrhoea present, n (%)	23 (74.2)	14 (53.8)
Median EDSS score (range)	1.5 (0.0–7.5) [*n* = 27]^[h]^	3.5 (0.0–7.0)
Median Rankin scale score (range)	1.0 (0.0–4.0) [*n* = 27]^[h]^	2.0 (0.0–4.0)

[a] Data not included due to missing data in 32 patients. [b] In the NL study, patients were split into subpopulations based on their age at treatment initiation (< 21 or ≥ 21 years). [c] Two patients were deceased when the NL retrospective study was initiated. [d] The total numbers of patients are based on the evaluable population in the NL (n=31) and IT (n=26) studies, unless otherwise stated. [e] Two patients had “elevated” levels and data were missing for two patients. [f] Data not reported. [g] Data were missing for 10 patients. [h] Data were missing for four patients. Abbreviations: EDSS, Expanded Disability Status Scale; IT, Italy; NL, the Netherlands; SD, standard deviation

#### Effectiveness

**Fig. 2:**
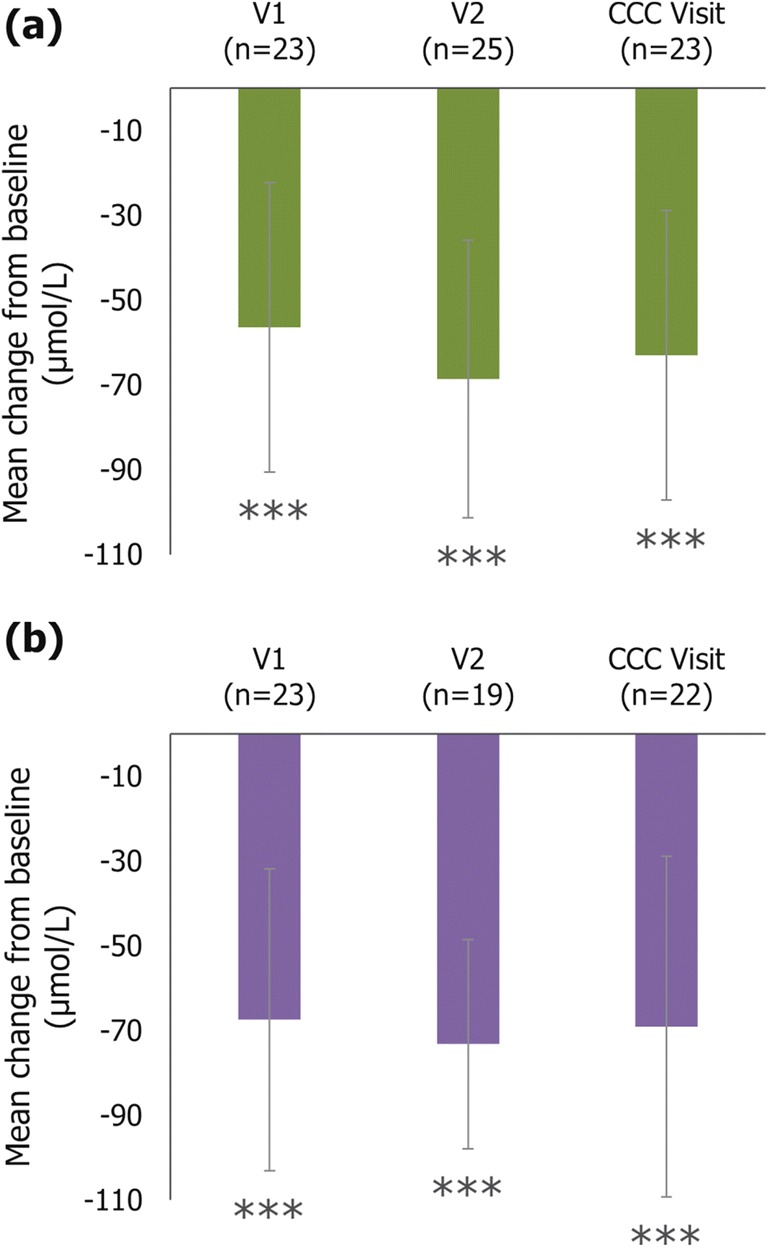
Mean change from baseline in serum cholestanol level (evaluable populations) (**a**) NL Study (*N*=31) (**b**) IT Study (*N*=26). Error bars represent the 95% confidence intervals; ***p < 0.001. The Italian study originally used values in mg/dL, which have been converted to μmol/L here. The original mean values (95% CI) for each visit in this study were: V1, -2.620 mg/dL (-3.313, -1.928); V2, -2.844 mg/dL (-3.324, -2.365); CCC Visit, -2.683 mg/dL (-3.463, -1.902). Abbreviations: CCC: Current clinical condition; IT: Italy; NL: the Netherlands; V1: Post-treatment visit 1; V2: Post-treatment visit 2

Mean serum cholestanol levels significantly decreased from baseline to any post-treatment visit (*p* < 0.001 at any visit; Fig. 2a). This was consistent between patients aged < 21 years or ≥ 21 years at treatment initiation (*p* < 0.001 at any visit; data not shown).

Neurological impairment was present in 64.5% (20/31) evaluable patients at baseline, 54.8% (17/31) at V1, 61.3% (19/31) at V2, and 54.8% (17/31) at the CCC visit. Psychiatric impairment resolved, improved or stabilised in the majority of patients with impairment at baseline (n=5/6; 85.7%). Of 31 evaluable patients, cognitive impairment was present in 58.1% (n=18/31) at baseline. At V1 and V2, there was improvement or stabilisation in 88.9% (n=16/18) of these patients (data missing for 1 patient), and by the CCC visit, cognitive symptoms had resolved, stabilised or improved in all 18 patients with impairment at baseline. Epilepsy resolved, and polyneuropathy stabilised or improved, in all patients with these symptoms at baseline (n=3 and 11, respectively). In addition, diarrhoea had resolved by the CCC visit in all patients who reported the symptom at the baseline visit (n=23; 100%).

Of the 26 patients with Rankin Scale assessments at both baseline and the CCC visit, there was an improvement in 15.4% (n=4), stabilisation in 69.2% (n=18) and deterioration in 15.4% (n=4) patients, although there was no difference in median Rankin Scale score from baseline to the CCC visit (0.0; range: -1–2). In addition, the median change from baseline in Rankin Scale score from baseline to the CCC visit was 0.0 (range: -0.1–0.0) in patients aged < 21 years at first treatment and 0.0 (range: 0.0–2.0) in patients aged ≥ 21 years at first treatment.

Of the 26 patients with EDSS scores at baseline and the CCC visit, there was an improvement in 23.1% (n=6), stabilisation in 53.8% (n=14) and deterioration in 23.1% (n=6) patients. The median change from baseline in EDSS score was 0.0 at any post-treatment visit (including when stratified by age at first treatment).

At baseline, all 29 patients with measurements for urinary bile alcohols had elevated levels. These decreased or were normalised in the majority of patients at all post-treatment visits (Supplemental Table 1, Online Resource). No significant difference was observed between the two patient subgroups (data not shown).

#### Safety

The median duration of CDCA treatment in the safety population was 9.00 (range: 0.4–26.3) years. A total of 76 AEs were reported in 26 (74.3%) patients (Table 2), including 9 serious AEs in 7 patients (20.0%), none of which were fatal or treatment-related. A full listing of AEs and serious AEs can be found in Supplemental Table 2 (Online Resource). Three patients had treatment-related AEs (two cases of constipation and one of toxic hepatitis); none were serious, or of severe intensity. The hepatitis case was an infant and has previously been reported by Huidekoper et al. (2016) [15]. Treatment was suspended and the event considered treatment-related, although hyperbilirubinaemia was present prior to initiation of treatment. Hepatitis did not recur on re-challenge and ongoing treatment with CDCA at a lower dose of 5 mg/kg/day.

**Table 2:** Summary of adverse events (safety populations)

	NL study (*N* = 35)		IT study (*N* = 28)	
	*n* (%)	Number of events	*n* (%)	Number of events
Any AEs (overall)	26 (74.3)	76	9 (32.1)	16
Mild AEs	23 (65.7)	53	1 (3.6)	1
Moderate AEs	15 (42.9)	22	5 (17.9)	6
Severe AEs	1 (2.9)	1	5 (17.9)	9
Discontinuations due to AEs	–	–	1 (3.6)	1
Treatment-related AEs	3 (8.6)	3	0 (0.0)	0
Serious AEs	7 (20.0)	9	9 (32.1)	15

Abbreviations: AE, adverse event; IT, Italy; NL, the Netherlands

### The IT study

#### Patient disposition and baseline characteristics

Twenty-eight patients were screened and enrolled in the study, all of whom were included in the safety analyses and 26 (92.9%) in the effectiveness analyses (Fig. 1). The gender split was approximately equal (46.4% male), and the mean (± SD) age of patients was 47.4 (± 13.2) years (Table 1). The median time from baseline visit to V1 was 23.7 months (range: 5.0–91.1), to V2 was 52.1 months (17.9–107.1), and to the CCC visit was 76.1 months (6.4–300.5).

#### Effectiveness

**Fig. 3:**
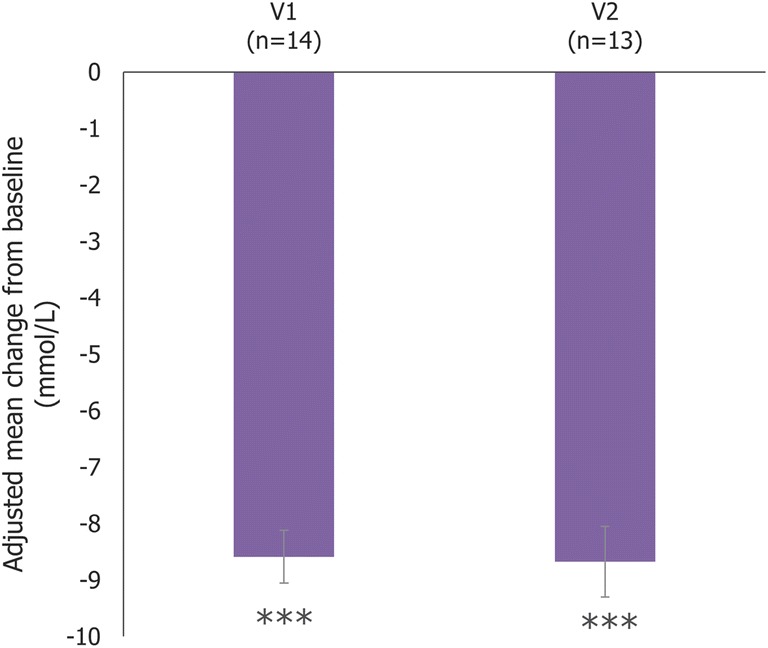
Mean change from baseline in 7α-hydroxy-4-cholesten-3-one level (IT study only; evaluable population). Error bars represent the 95% confidence intervals; ***p < 0.001. The Italian study originally used values in μg/dL, which have been converted to μmol/L here. The original mean values (95% CI) for each visit in this study were: V1, -343.92 μg/dL (-353.27, -334.58); V2, -347.53 μg/dL (-360.08, -334.98). Abbreviations: IT: Italy; V1: Post-treatment visit 1; V2: Post-treatment visit 2

Mean serum cholestanol levels significantly decreased from baseline to any post-treatment visit (*p* < 0.001 at any visit; Fig. 2b). Furthermore, the mean levels of 7α-hydroxy-4-cholesten-3-one significantly decreased from baseline to V1 and V2 (*p* < 0.001; Fig. 3).

Neurological impairment was present in 20 patients (76.9%) at baseline and had stabilised in 9 (45.0%) and deteriorated in 11 (55.0%) of these patients at the CCC visit. Of the 13 patients who presented with psychiatric impairment at baseline, 92.3% (n=12) improved or remained stable at the CCC visit. Of 26 evaluable patients, cognitive impairment was present in 20 patients (76.9%) at baseline and stabilised in 16 (72.7%) patients at the CCC visit. In addition, diarrhoea stabilised, improved or disappeared by the CCC visit in the majority of patients who had this symptom at baseline (n=13/14; 92.8%).

There was no deterioration in Rankin Scale score in 61.5% (n=16/26) patients for the entire follow-up period, and there was no difference in the median Rankin Scale score from baseline to the CCC visit (0.0; range: 0–2). Of 26 evaluable patients, EDSS scores remained stable in 46.1% (n=12/26) patients, improved in 3.8% (n=1/26) patients and deteriorated in 50.0% (n=13/26) patients during the study. The median changes from baseline in EDSS scores were 0.0 at V1 (range: 0.0–2.5) and V2 (range: -0.5–2.0), and 0.5 (range: -0.5–4.0) at the CCC visit.

#### Safety

The median duration of treatment in the safety population was 5.75 (range: 0.0–25.0) years. A total of 16 AEs were reported in 9 patients (32.1%), including 15 serious AEs in 9 patients (32.1%; Table 2). The latter included one fatal case of colon cancer. A full listing of AEs and serious AEs can be found in Supplemental Table 2 (Online Resource). Severe AEs were reported in 5 patients (17.9%), although no patients had treatment-related AEs.

## Discussion

CTX is a rare neurometabolic disease that results in neurodegeneration, and for which CDCA treatment is the standard of care [4]. The two retrospective studies presented here investigated the safety and effectiveness of CDCA in two cohorts of patients with confirmed CTX and ≥ 1 year of exposure to the drug. Across both studies, CDCA was generally effective and well-tolerated, with improvement, resolution or stabilisation of the signs and symptoms of disease in most patients. In addition, there was some evidence of improvement or stabilisation in disability scale scores, although a number of patients also showed deterioration in these measures. Improvements in the levels of biochemical markers were evident in both cohorts, including serum cholestanol, 7α-hydroxy-4-cholesten-3-one, and/or urinary bile alcohols. These outcomes reflect those seen in previous studies of CDCA treatment in patients with CTX [8, 14, 16–18].

Though these observations were broadly comparable between the two studies, there were some differences. For instance, while symptoms such as diarrhoea and neurological, psychiatric and cognitive impairment frequently improved or resolved in the NL study, they generally remained stable in the IT study. Differences in age at diagnosis and treatment initiation may be important reasons for this (patients in the IT study were older and diagnosed later in life on average than those in the NL study), although product differences cannot be ruled out. The observation that mean serum cholestanol levels significantly declined at each visit, in both cohorts, demonstrates the comparable effectiveness of the different formulations and suggests that demographic variation underlies the apparent differences in treatment effectiveness. Inter-study differences were also observed in the Rankin Scale and EDSS scores; this might also be attributable to the older age of patients in the IT study and the greater severity of their symptoms at baseline. Differences between the two studies could also have arisen due to assessment and investigator variabilities, such as in the physical examinations conducted to assess disease signs and symptoms.

In addition to differences in the clinical outcomes, the rate of reported AEs was lower in the IT cohort than the NL cohort. In both studies, AEs were documented and collected on the basis of source documents available at the site, and it is not known whether the differences we report here are real or due to variations in reporting habits. The NL study also included more AEs unrelated to CDCA treatment; however, it remains unclear whether such events were either not reported or not recorded in the IT study.

Beyond the usual limitations associated with retrospective data collection (e.g. missing records and the absence of a control group), a specific limitation of these studies included the use of raw EDSS scores rather than EDSS thresholds, which usually range from 0 to 10 in increments of 0.5. This means that while improvement in EDSS score was observed for some patients, it is difficult to discern whether these improvements were clinically significant across the evaluable population. Nevertheless, given the chronic and progressive nature of CTX, many patients already have advanced disease at the time of diagnosis, and thus stabilisation of symptoms can be as important as improvement. An additional limitation of these studies was the exclusion of patients in the Netherlands who were institutionalised and therefore unable to attend hospital visits. It is not clear to what extent this might have impacted the observed outcomes.

In summary, the results of these retrospective cohort studies indicate that CDCA is generally effective at reducing cholestanol levels and stabilising or improving the progression of disability and most neurological and non-neurological manifestations. These studies also demonstrate that CDCA has an acceptable safety profile in CTX patients undergoing treatment for a prolonged period of time.

## Electronic supplementary material

ESM 1 (PDF 115 kb)
