# Supplementary material for: The safety and effectiveness of chenodeoxycholic acid treatment in patients with cerebrotendinous xanthomatosis: two retrospective cohort studies
Source: Neurol Sci. 2019 Dec 20;41(4):943–9. doi: 10.1007/s10072-019-04169-8 (PMC7160076; doi:10.1007/s10072-019-04169-8)
Supplement: Supplementary file 1 — (PDF 115 kb) [file 10072_2019_4169_MOESM1_ESM.pdf]

The Safety and Effectiveness of Chenodeoxycholic Acid Treatment in Patients with Cerebrotendinous Xanthomatosis: Two Retrospective Cohort Studies

Neurological Sciences

Verrips A,[1] Dotti MT,[2] Mignarri A,[2] Stelten BML,[3] Verma S,[4] Federico A[2]

[1]Canisius-Wilhelmina Hospital, Nijmegen, The Netherlands; [2]Department of Medicine, Surgery and Neuroscience, University of Siena, Siena, Italy; [3]Catharina Hospital, Eindhoven, The Netherlands; [4]Leadiant Biosciences Ltd., London, United Kingdom.

Correspondence to: Dr Aad Verrips (A.Verrips@cwz.nl; +31-24-3658765)

SUPPLEMENTAL MATERIAL

Statistical Analyses (Both Studies)

Descriptive statistics are presented, including patient number (n) and frequency (%), arithmetic mean, 95% confidence intervals (95% CI), and median. Missing values were included in the denominator count when computing percentages. Changes from baseline at V1, V2 and CCC visit were evaluated using a paired t-test, and statistical significance declared if the rounded p-value was ≤0.05. In the NL study, sub-analyses were conducted based on patients' age at treatment initiation (<21 or ≥21 years), as studies have demonstrated differences in outcomes depending on age at diagnosis and at treatment initiation [1].

The analysis of 7α-hydroxy-4-cholesten-3-one was based on a mixed model for repeated measures, with change from baseline in 7α-hydroxy-4-cholesten-3-one at V1 and V2 as dependent variables, visit as a fixed effect, and the baseline and baseline × visit interaction as covariates. An unstructured covariance matrix was considered, and the Kenward-Roger adjustment was used for the degrees of freedom.

Supplemental Table 1. Urinary bile alcohols (NL study only; evaluable population)

PT Visits	Baseline Visit				
**n (%)**	**Elevated**	**Mildly elevated**	**Normal**	**Missing/not done**	**Total**
**V1 (n=31)**					
**Elevated**	3 (9.7)	0 (0.0)	0 (0.0)	0 (0.0)	3 (9.7)
**Mildly elevated**	12 (38.7)	0 (0.0)	0 (0.0)	0 (0.0)	12 (38.7)
**Normal**	6 (19.4)	0 (0.0)	0 (0.0)	0 (0.0)	6 (19.4)
**Missing/not done**	8 (25.8)	0 (0.0)	0 (0.0)	2 (6.5)	10 (32.3)
**Total**	29 (93.5)	0 (0.0)	0 (0.0)	2 (6.5)	31 (100.0)
p-value (Test of symmetry) <0.001					
**V2 (n=31)**					
**Elevated**	0 (0.0)	0 (0.0)	0 (0.0)	0 (0.0)	0 (0.0)
**Mildly elevated**	7 (22.6)	0 (0.0)	0 (0.0)	0 (0.0)	7 (22.6)
**Normal**	10 (32.3)	0 (0.0)	0 (0.0)	2 (6.5)	12 (38.7)
**Missing/not done**	12 (38.7)	0 (0.0)	0 (0.0)	0 (0.0)	12 (38.7)
**Total**	29 (93.5)	0 (0.0)	0 (0.0)	2 (6.5)	31 (100.0)
p-value (Test of symmetry) <0.001					
**CCC visit (n=31)**					
**Elevated**	3 (9.7)	0 (0.0)	0 (0.0)	0 (0.0)	3 (9.7)
**Mildly elevated**	4 (12.9)	0 (0.0)	0 (0.0)	0 (0.0)	4 (12.9)
**Normal**	6 (19.4)	0 (0.0)	0 (0.0)	1 (3.2)	7 (22.6)
**Missing/not done**	16 (51.6)	0 (0.0)	0 (0.0)	1 (3.2)	17 (54.8)
**Total**	29 (93.5)	0 (0.0)	0 (0.0)	2 (6.5)	31 (100.0)
p-value (Test of symmetry) <0.001					

Abbreviations: CCC: Current clinical condition; NL: The Netherlands; V1: Post-treatment visit 1; V2: Post-treatment visit 2.

Supplemental Table 2. Adverse events and serious adverse events (safety populations)

	NL Study (N=35)		IT Study (N=28)	
	n (%)	Number of events	n (%)	Number of events
**Any AE**	26 (74.3)	76	9 (32.1)	16
**Surgical and medical procedures**	11 (31.4)	18	6 (21.4)	9
**Musculoskeletal and connective tissue disorders**	6 (17.1)	7	-	-
**Gastrointestinal disorders**	5 (14.3)	7	2 (7.1)	2
**General disorders and administration site conditions**	5 (14.3)	5	-	-
**Injury, poisoning and procedural complications**	5 (14.3)	5	1 (3.6)	1
**Cardiac disorders**	4 (11.4)	5	-	-
**Skin and subcutaneous tissue disorders**	4 (11.4)	4	-	-
**Hepatobiliary disorders**	3 (8.6)	3	1 (3.6)	1
**Infections and infestations**	3 (8.6)	4	-	-
**Metabolism and nutrition disorders**	3 (8.6)	3	-	-
**Nervous system disorders**	3 (8.6)	3	-	-
**Eye disorders**	2 (5.7)	2	1 (3.6)	1
**Immune system disorders**	2 (5.7)	2	-	-
**Psychiatric disorders**	2 (5.7)	2	-	-
**Respiratory, thoracic and mediastinal disorders**	2 (5.7)	2	-	-
**Vascular disorders**	2 (5.7)	2	-	-
**Investigations[a]**	1 (2.9)	1	-	-
**Renal and urinary disorders**	1 (2.9)	1	-	-
**Neoplasms benign, malignant and unspecified (including cysts and polyps)**	-	-	1 (3.6)	1
**Pregnancy, puerperium and perinatal conditions**	-	-	1 (3.6)	1
**Any Serious AE**	7 (20.0)	9	9 (32.1)	15
**Surgical and medical procedures**	3 (8.6)	3	6 (21.4)	9

**Gastrointestinal disorders**	-	-	2 (7.1)	2
**Cardiac disorders**	1 (2.9)	1	-	-
**Eye disorders**	-	-	1 (3.6)	1
**Injury, poisoning and procedural complications**	1 (2.9)	1	1 (3.6)	1
**Musculoskeletal and connective tissue disorders**	1 (2.9)	1	-	-
**Psychiatric disorders**	1 (2.9)	1	-	-
**Renal and urinary disorders**	1 (2.9)	1	-	-
**Respiratory, thoracic and mediastinal disorders**	1 (2.9)	1	-	-
**Neoplasms benign, malignant and unspecified (including cysts and polyps)**	-	-	1 (3.6)	1
**Pregnancy, puerperium and perinatal conditions**	-	-	1 (3.6)	1

[a] Weight increased. Patients are only counted once for each row in the column “n (%)” and percentages are calculated based on the number of patients (N). Abbreviations: IT: Italy; NL: The Netherlands.

REFERENCE

1. Yahalom G, Tsabari R, Molshatzki N, Ephraty L, Cohen H, Hassin-Baer S (2013) Neurological outcome in cerebrotendinous xanthomatosis treated with chenodeoxycholic acid: early versus late diagnosis. Clinical neuropharmacology 36 (3):78-83. doi:10.1097/WNF.0b013e318288076a
